# Characterization of the VP2 and NS1 genes from canine parvovirus type 2 (CPV-2) and feline panleukopenia virus (FPV) in Northern China

**DOI:** 10.3389/fvets.2022.934849

**Published:** 2022-11-28

**Authors:** Shaohan Li, Xin Chen, Yunfeng Hao, Guangzhi Zhang, Yanli Lyu, Jianke Wang, Weiquan Liu, Tong Qin

**Affiliations:** ^1^Institute of Animal Sciences, Chinese Academy of Agricultural Sciences, Beijing, China; ^2^State Key Laboratory of Agrobiotechnology, Department of Biochemistry and Molecular Biology, College of Biological Sciences, China Agricultural University, Beijing, China; ^3^College of Veterinary Medicine, Hebei Agricultural University, Baoding, China

**Keywords:** canine parvovirus type 2, feline panleukopenia virus, epidemiology, genetic characterization, phylogenetic analysis

## Abstract

Canine parvovirus type 2 (CPV-2) and feline panleukopenia virus (FPV) cause severe disease in young animals, pups, and kittens. CPV-2 evolved from FPV by altering the species-specific binding of the viral capsid to the host receptor, i.e., the transferrin receptor (TfR), and CPV-2 genetic variants have been identified by specific VP2 amino acid residues (297, 426). Early studies focused on the main capsid protein VP2; however, there have been limited studies on the non-structural protein NS1. In this study, we identified the genetic variants of clinical samples in dogs and cats in northern China during 2019–2020. The genetic characterization and phylogenetic analyses of VP2 and NS1 gene were also conducted. The results revealed that the CPV-2c was identified as the major genetic variant. One new CPV-2b and two CPV-2c strains were collected from cats. Four mutation sites (60, 630, 443, and 545 amino acid residues) were located in the functional domains of the NS1 protein. The phylogenetic analysis of VP2 and NS1 genes showed that they were clustered by geographical regions and genotypes. The gene mutation rate of CPV-2 was increasing in recent years, resulting in a complex pattern of gene evolution in terms of host preference, geographical selection, and new genetic variants. This study emphasizes that continuous molecular epidemiological surveillance is required to understand the genetic diversity of FPV and CPV-2 strains.

## Introduction

Canine parvovirus type 2 (CPV-2) and feline panleukopenia virus (FPV) infections seriously threaten the health of dogs and cats, especially puppies and kittens, with high morbidity and mortality rates (1, 2). FPV was identified at the beginning of the 20th century and has maintained a certain degree of genetic stability (3). CPV-2 was first reported in the USA and Australia in 1978 (4). The emergence of CPV-2 resulted from site-specific mutations on the surface of the viral capsid, and the cross-species transmission of CPV-2 occurs by altering the species-specific binding of the viral capsid to the host receptor, i.e., the transferrin receptor TfR (5, 6). Although CPV-2 is a DNA virus, the rate of amino acid mutations almost reaches that of RNA viruses (7). After its emergence, a new antigenic variant (CPV-2a) emerged with five important amino acid mutations (M87L, I101T, A300G, D305Y, and V555I) in the VP2 gene from 1979 to 1981. These mutations affected the antigenic reactivity of monoclonal antibodies, the binding affinity to TfR, and the ability to replicate in cats (8). In 1984, another antigenic variant (CPV-2b) emerged, with two amino acid mutations (N426D and I555V). A third genetic variant (CPV-2c), with 426E, was reported in 2000. At the end of the 20th century, the new CPV-2a and new CPV-2b genetic variants were identified, carrying an S297A substitution (9). In the 2000s, the CPV-2c genetic variant was frequently found in Europe and South America but was relatively rare in Asia, where CPV-2a was more predominant (10). However, currently, the CPV-2c genetic variant is distributed worldwide including in Asia, Africa, Australia, Europe, North and South America, China, Vietnam, Mongolia, and Thailand (11). In China, the molecular surveillance carried out between 1986 and 2017 showed a clear predominance of CPV-2a. However, CPV-2c strains had become dominant in 2018 and 2021. One CPV-2c strain with typical amino acid residues (5G, 267Y, 324I, and 370R) in the VP2 protein has been recognized as the “Asian CPV-2c” genetic variant, which had never been reported prior to 2013 (12). The reasons for the emergence of different antigenic variants and their spread among different hosts have been attributed to amino acid mutation sites (13, 14).

CPV-2 and FPV are non-enveloped single-stranded linear DNA viruses of the *Parvoviridae* family (15), with a genome of ~5,200 nt in length (16). The genome contains two open reading frames (ORFs), which encode for two non-structural (NS1 and NS2) and two structural (VP1 and VP2) proteins (17). VP2 protein, the main component of the viral capsid, not only induces the neutralizing antibodies by acting as the main protective antigen, but also determines hemagglutination, cellular tropism, and the host ranges of the virus (18). Additionally, the NS1 protein plays an important role in virus replication, DNA packaging, cytotoxicity, and pathogenicity (18–20). In this study, we aimed to perform molecular and phylogenetic analyses of the VP2 and NS1 genes from FPV and CPV-2 variants in northern China.

## Materials and methods

### Clinical samples

A total of 84 samples (feces or rectal swabs) were collected from domestic dogs (*n* = 51) and cats (*n* = 33) with clinical signs, like fever, vomiting, and diarrhea. Animals were initially identified to be infected with CPV-2 or FPV using colloidal gold test strips (BioNote Rapid Test Kit; BioNote, Gyeonggi-do, Korea). The samples were collected from different areas of northern China: Xiong'an (*n* = 31), Jinan (*n* = 8), Langfang (*n* = 1), Haidian District in Beijing (*n* = 4), and Tongzhou District in Beijing (*n* = 40). Viral DNA was extracted by the Aidlab DNA rapid extraction kit (Aidlab Biotech, Beijing, China), according to the manufacturer's instructions, and stored at −20°C until further processing. The details are summarized in Table 1.

**Table 1:** Detailed description of canine parvovirus type 2 (CPV-2) and feline panleukopenia virus (FPV) characterized in this study.

**Strains**	**Origin**	**Species**	**NS1**	**VP2**	**Strains**	**Origin**	**Species**	**NS1**	**VP2**	**Strains**	**Origin**	**Species**	**NS1**	**VP2**
XA-CPV2c-0-1	Xiongan	Dog	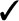	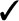	XA-CPV2c-0-18	Xiongan	Dog	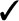	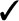	HD-CPV2c-23	Haidian	Dog	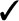	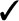
XA-newCPV2a-0-2	Xiongan	Dog	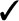	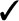	XA-CPV2c-0-19	Xiongan	Dog	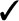	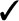	TZ-CPV2c-126	Tongzhou	Dog	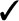	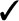
XA-CPV2c-0-3	Xiongan	Dog	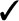	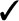	XA-CPV2c-0-20	Xiongan	Dog	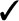	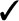	TZ-newCPV2a-127	Tongzhou	Dog	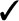	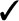
XA-newCPV2a-0-4	Xiongan	Dog	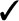	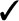	XA-CPV2c-0-21	Xiongan	Dog	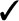	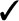	TZ-CPV2c-131	Tongzhou	Dog	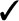	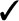
XA-newCPV2a-0-5	Xiongan	Dog	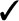	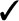	XA-CPV2c-0-22	Xiongan	Dog	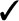	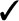	TZ-newCPV2a-132	Tongzhou	Dog	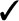	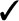
XA-CPV2c-0-6	Xiongan	Dog	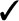	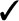	XA-CPV2c-0-23	Xiongan	Dog	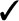	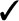	TZ-CPV2c-138	Tongzhou	Dog	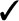	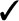
XA-CPV2c-0-7	Xiongan	Dog	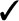	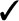	XA-newCPV2a-1	Xiongan	Dog	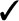	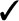	TZ-CPV2c-141	Tongzhou	Dog	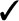	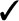
XA-CPV2c-0-8	Xiongan	Dog	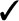	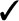	XA-newCPV2a-2	Xiongan	Dog	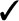	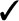	TZ-newCPV2a-142	Tongzhou	Dog	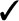	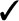
XA-newCPV2a-0-9	Xiongan	Dog	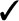	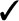	XA-newCPV2a-3	Xiongan	Dog	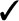	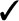	TZ-newCPV2a-149	Tongzhou	Dog	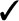	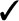
XA-CPV2c-0-10	Xiongan	Dog	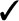	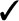	XA-CPV2c-4	Xiongan	Dog	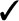	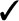	TZ-newCPV2b-150	Tongzhou	Dog	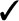	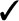
XA-CPV2c-0-11	Xiongan	Dog	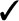	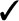	XA-CPV2c-5	Xiongan	Dog	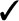	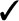	TZ-CPV2c-152	Tongzhou	Dog	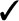	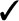
XA-CPV2c-0-12	Xiongan	Dog	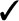	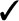	XA-newCPV2a-6	Xiongan	Dog	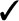	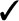	TZ-CPV-158	Tongzhou	Dog	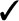	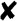
XA-CPV2c-0-13	Xiongan	Dog	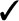	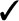	XA-CPV-7	Xiongan	Dog	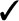	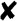	TZ-CPV2c-164	Tongzhou	Dog	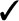	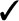
XA-CPV2c-0-14	Xiongan	Dog	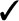	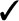	XA-CPV2c-8	Xiongan	Dog	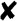	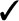	TZ-CPV2c-165	Tongzhou	Dog	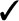	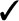
XA-CPV2c-0-15	Xiongan	Dog	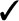	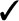	JN-CPV2c-9	Jinan	Dog	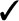	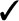	TZ-CPV2c-171	Tongzhou	Dog	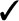	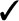
XA-CPV2c-0-16	Xiongan	Dog	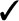	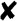	JN-newCPV2a-10	Jinan	Dog	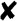	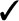	TZ-CPV2c-173	Tongzhou	Dog	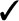	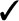
XA-CPV2c-0-17	Xiongan	Dog	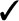	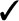	HD-CPV-15	Jinan	Dog	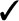	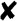	TZ-CPV2c-174	Tongzhou	Dog	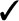	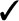
JN-FPV-11	Jinan	Cat	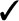	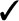	TZ-FPV-119	Tongzhou	Cat	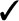	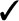	TZ-FPV-235	Tongzhou	Cat	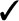	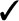
JN-FPV-12	Jinan	Cat	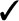	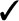	TZ-cat-CPV2c-121	Tongzhou	Cat	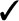	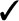	TZ-FPV-236	Tongzhou	Cat	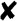	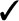
JN-FPV-87	Jinan	Cat	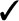	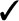	TZ-FPV-122	Tongzhou	Cat	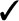	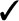	TZ-FPV-237	Tongzhou	Cat	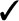	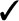
JN-FPV-90	Jinan	Cat	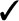	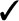	TZ-FPV-124	Tongzhou	Cat	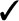	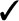	TZ-FPV-238	Tongzhou	Cat	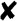	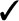
JN-FPV-91	Jinan	Cat	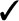	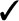	TZ-FPV-133	Tongzhou	Cat	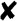	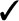	TZ-FPV-239	Tongzhou	Cat	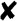	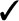
JN-FPV-92	Jinan	Cat	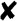	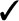	TZ-FPV-135	Tongzhou	Cat	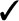	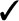	TZ-FPV-240	Tongzhou	Cat	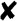	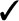
JN-FPV-96	Jinan	Cat	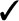	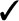	TZ-FPV-143	Tongzhou	Cat	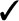	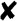	TZ-FPV-241	Tongzhou	Cat	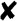	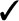
TZ-FPV-99	Tongzhou	Cat	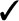	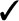	TZ-FPV-148	Tongzhou	Cat	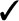	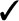	TZ-FPV-242	Tongzhou	Cat	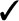	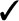
TZ-FPV-104	Tongzhou	Cat	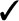	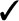	TZ-FPV-185	Tongzhou	Cat	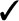	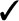	TZ-FPV-243	Tongzhou	Cat	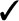	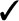
TZ-FPV-108	Tongzhou	Cat	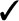	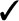	TZ-FPV-193	Tongzhou	Cat	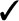	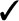	HD-cat-newCPV2b-9	Haidian	Cat	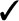	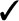
TZ-FPV-112	Tongzhou	Cat	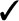	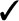	TZ-FPV-195	Tongzhou	Cat	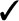	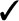	HD-cat-CPV2c-79	Haidian	Cat	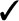	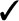

“✓” indicates successful sequencing; “
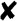
” indicates sequencing failure; NS1, non-structural protein 1; VP2, structural protein.

The principle of virus naming is based on the “abbreviation of region name-virus genotype-number.”

### PCR and DNA sequencing assays

The clinical positive samples were further confirmed by PCR, targeting the 573 bp and 286 bp VP2 fragments of CPV-2 and FPV, respectively (Table 2). PCR was performed using PrimeSTAR^®^ Max DNA Polymerase (Takara Biotech, Dalian, China) according to the manufacturer's instructions, in a final volume of 20 μl comprising 10 μl of PrimeSTAR MAX Premix (2 × ), 1 μl of each primer (10 μM), 1 μl of DNA, and 7 μl of sterile distilled water. The PCR condition was set as an initial denaturation step at 98°C for 1 min; 35 cycles at 98°C for 10 s, annealing at 56°C for 5 s, extension at 72°C for 5 s; and a final extension at 72°C for 10 min. The positive samples were then subjected to the amplification of the complete NS1 and VP2 genes using two primer pairs NS1-F/R or VP2-F/R, respectively, in a final volume of 50 μl, comprising 25 μl of PrimeSTAR MAX Premix (2 × ), 1 μl of each primer (10 μM), 2 μl of DNA, and 21 μl of sterile distilled water. The PCR condition was set as an initial denaturation step at 98°C for 1 min; 35 cycles at 98°C for 10 s, annealing at 57°C for 15 s, extension at 72°C for 30 s; and a final extension at 72°C for 10 min. Positive amplicons were purified using a gel extraction kit (OMEGA Bio Tek, Winooski, VT, USA) and submitted to BGI biotech in Beijing for direct Sanger sequencing. The VP2 gene was sequenced using the primers pair used for amplification (VP2-F/R) and with three additional internal primers (CPV-VP2-F/R/F1). The NS1 gene was also sequenced using the primers pair used for amplification (NS1-F/R) and with three additional internal primers (CPV-NS1-658F/800R/1228F). Detailed information on the primers is shown in Table 2. Consensus sequences were assembled using the EditSeq program (DNAStar, Madison, WI, USA).

**Table 2:** Primers used in this study.

**Name**	**Sequence (5^′^-3^′^)**	**^d^ Location (bp)**	**Size (bp)**
CPV-F	TGATGGAGCAGTTCAACCAGA	2789–2809	573 bp^a^
CPV-R	TCAGATCTCATAGCTGCTGGA	3341–3361	
FPV-F	AGCTACAGGATCTGGGAACG	2840–2859	286 bp^a^
FPV-R	TGCATCAACCAATGACCAAGG	3105–3125	
VP2-F	GGCGAATTCATGAGTGATGGAGCAGTTC	2775–2802	1770 bp^b^
VP2-R	CGCCTCGAGATATAATTTTCTAGGTGCT	4517–4544	
NS1-F	GACCGTTACTGACATTCGC	208–226	2259 bp^b^
NS1-R	CGGCGTCAGAAGGGTT	2451–2466	
CPV-VP2-F	CTATGCCATTTACTCCAG	3328–3345	//^c^
CPV-VP2-R	CCTGTTCTTAGTAAGTG	3540–3556	
CPV-VP2-F1	CTACCACAACAGGAGAAACACC	3946–3967	
CPV-NS1-658F	GTCCACATGACAAAAGAAAG	927–946	//^c^
CPV-NS1-800R	CTTTGTTTCCTGTGCTGTCG	1069–1088	
CPV-NS1-1228F	GCTCAAGCCATAGCACAAGC	1497–1516	

a CPV-F/R and FPV-F/R used for viral screening.

b VP2-F/R and NS1-F/R were used for amplification for Sanger sequencing.

c CPV-VP2-F/R/F1 and CPV-NS1-658F/800R/1228F were used as internal primers for the Sanger sequence.

d The primers were designed according to a reference sequence (GenBank: MH545963.1).

### Sequence and phylogenetic analyses

The complete coding sequences of the NS1 and VP2 genes were assembled and translated into amino acid sequences (668 and 584 aa, respectively) using the EditSeq software. The translated VP2 and NS1 amino acid sequences were aligned with reference sequences (new CPV-2a, AY742935; new CPV-2a, EF011664; new CPV-2a, JN867611; new CPV-2b, EU659119; new CPV-2b, JQ268284; new CPV-2b, LC270892; CPV-2c, KM457125; CPV-2c, KU508407; CPV-2c, MK388674; CPV-2c, MN832850; FPV, KP019621; FPV, MT614366), which were obtained from the NCBI database. The sequence alignments were obtained using the ClustalW program included in the MegAlign software (DNAStar).

The phylogenetic relationships were evaluated using MEGA6.0 (21) and iTOL software (22). The phylogenetic trees were constructed using the neighbor-joining and maximum-likelihood methods. A bootstrap analysis with 1,000 replicates was performed to assess the confidence level of the branching pattern. For a more comprehensive evaluation, 41 sequences of the NS1 gene and 46 sequences of the VP2 gene were downloaded from GenBank as reference sequences (Supplementary Table 1).

## Results

### Antigenic typing of FPV and CPV-2 variants

A total of 84 clinical samples were confirmed to be positive for FPV or CPV-2. Sequencing of five VP2 and nine NS1 gene sequences was not successful, as shown in Table 1. Based on 79 VP2 amino acid sequences, 32 sequences from cats and 47 sequences from dogs were obtained. Among the 47 strains from dogs, 13, 1, and 33 strains were typed as new CPV-2a, new CPV-2b, and CPV-2c, respectively. Twenty-nine strains from the 32 cats were typed as FPV, and one strain (HD-cat-newCPV2b-9) was identified as new CPV-2b and two strains (HD-cat-CPV-2c-79 and TZ-cat-CPV-2c-121) were identified as CPV-2c.

### Sequence analysis of VP2 gene

Sequence analysis showed that all CPV-2c strains had Arg at VP2-370 amino acid residue, except for XA-CPV-2c-0-8 which showed Gln. Meanwhile, new CPV-2a/2b strains showed Gln at the same amino acid residue. In addition, CPV-2c strains harbored 440Thr, and the new CPV-2a/2b strains contained 440Ala. Furthermore, other mutations also emerged among the CPV strains, i.e., P13S (TZ-CPV-164), E18D (XA-CPV-0-22), L218I (XA-CPV-0-14), and G441R (JN-CPV-10 and TZ-CPV-150). The VP2 gene of FPV strains was more conserved than that of CPV-2 strains. Indeed, 44.8% (13/29) of FPV strains showed the A91S mutation. Meanwhile, three other VP2 mutations were observed in FPV strains: F290Y (TZ-FPV-236), T425A (JN-FPV-12 and JN-FPV-90), and V535I (TZ-FPV-243).

### Sequence analysis of NS1 gene

Based on 75 NS1 amino acid sequences, 26 sequences from cats and 49 sequences from dogs were obtained. The mutation rates of the non-synonymous amino acid mutation sites in different virus types and genetic variants (FPV/new CPV-2a/new CPV-2b/CPV-2c) are shown in Table 3.

**Table 3:** Statistics of mutation sites and rates in the NS1 gene from FPV and different CPV-2 genetic variants.

**Amino acid**	**19**	**23**	**60**	**115**	**187**	**222**	**247**	**248**	**356**	**443**	**544**	**545**	**572**	**583**	**596**	**624**	**630**	**667**
**positions**																		
**Amino acid**	**K → R**	**N → D**	**I → V**	**V → I**	**I → V**	**M → I**	**Q → H**	**I → T**	**N → K**	**I → V**	**Y → F**	**E → K**	**E → K**	**E → K**	**V → L**	**N → K**	**L → P**	**L → V**
**mutations**												**E → V**						
FPV (total = 23)	//^a^	8	2	5	//	2	8	18	//	8	1	2 (K)	8	//	8	1	//	1
sample number																		
Mutation rate (%)	//	34.8	8.7	21.7	//	8.7	34.8	78.3	//	34.8	4.3	8.7	34.8	//	34.8	4.3	//	4.3
New CPV-2a (total = 12)	1	//	2	//	1	//	//	//	2	//	3	3 (V)	9	1	//	4	2	//
sample number																		
Mutation rate (%)	8.3	//	16.7	//	8.3	//	//	//	16.7	//	25.0	25.0	75.0	8.3	//	33.3	16.7	//
New CPV-2b (total = 2)	2	//	//	//	//	//	//	//	//	//	2	2 (V)	//	2	//	//	//	//
sample number																		
Mutation rate (%)	100	//	//	//	//	//	//	//	//	//	100	100	//	100	//	//	//	//
CPV-2c (total = 38)	//	//	30	//	1	2	//	//	3	//	31	31 (V)	6	1	//	2	30	1
sample number																		
Mutation rate (%)	//	//	78.9	//	2.6	5.3	//	//	7.9	//	81.6	81.6	15.8	2.6	//	5.3	78.9	2.6

“//” means no data in this table.

CPV-2 and FPV presented common mutation sites at amino acids 60, 222, 544, 545, 572, 624, and 667. Residues 247 and 545 were further analyzed due to their key role in protein function. All CPV-2 sequences showed 247Q, while 39.1% of FPV sequences showed 247R, and the remaining 60.9% of FPV sequences showed 247Q. Moreover, in this study, 8.7% of the FPV strains showed E545K, and 69.2% (36/52) of CPV-2 strains harbored E545V. Amino acid mutations 19, 187, 222, 356, 583, 596, 624, and 667 were newly discovered in this study, which awaited further investigation.

### Phylogenetic analysis of VP2 gene

**Figure 1:**
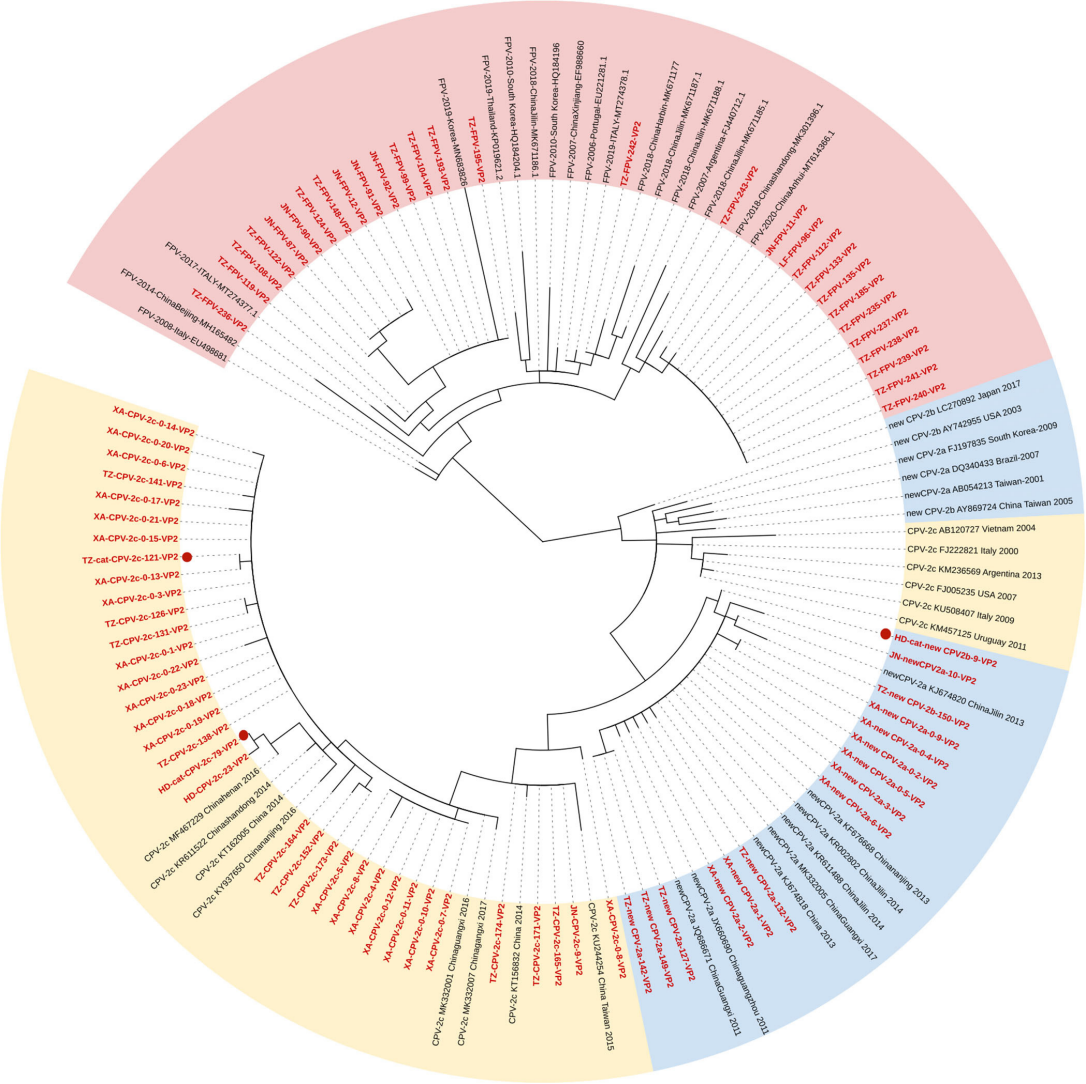
Phylogenetic tree based on a comparison of the structural (VP2) gene of canine parvovirus type 2 (CPV-2) and feline panleukopenia virus (FPV). Phylogenetic relationships were calculated using the maximum likelihood (ML) method. Gaps were handled by pairwise deletion and bootstrap values were calculated from 1,000 replicates. Strains analyzed in this study are marked in red and reference strains are marked in black. CPV-2 strains isolated from cats in this study are marked with “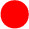.” The monophyletic clusters of new CPV-2a/2b are marked in blue (Clade IIA), the monophyletic clusters of CPV-2c are marked in yellow (Clade IIB), and the monophyletic clusters of FPV are marked in pink (Clade I).

Figure 1 shows the phylogenetic tree inferred from the VP2 gene, which clustered into two main clades (Clades I and II; FPV and CPV-2). Fifteen FPV strains were phylogenetically closed to foreign strains and 13 FPV strains were closed to the strains of Asian origin. All the CPV-2 sequences were clustered in Clade II, and the branches were formed by genetic types. Clade II could be further divided into two parts: Clade IIA clustering new CPV-2a/2b strains and Clade IIB clustering CPV-2c strains. The phylogenetic analysis showed that the new CPV-2b strain infecting cats (HD-cat-newCPV2b-9) was close to another new CPV-2b strain from dogs (XA-newCPV-2b-150). Meanwhile, two CPV-2c strains infecting cats (HD-cat-CPV-2c-79 and TZ-cat-CPV-2c-121) also clustered with other CPV-2c strains from dogs.

### Phylogenetic analysis of NS1 gene

**Figure 2:**
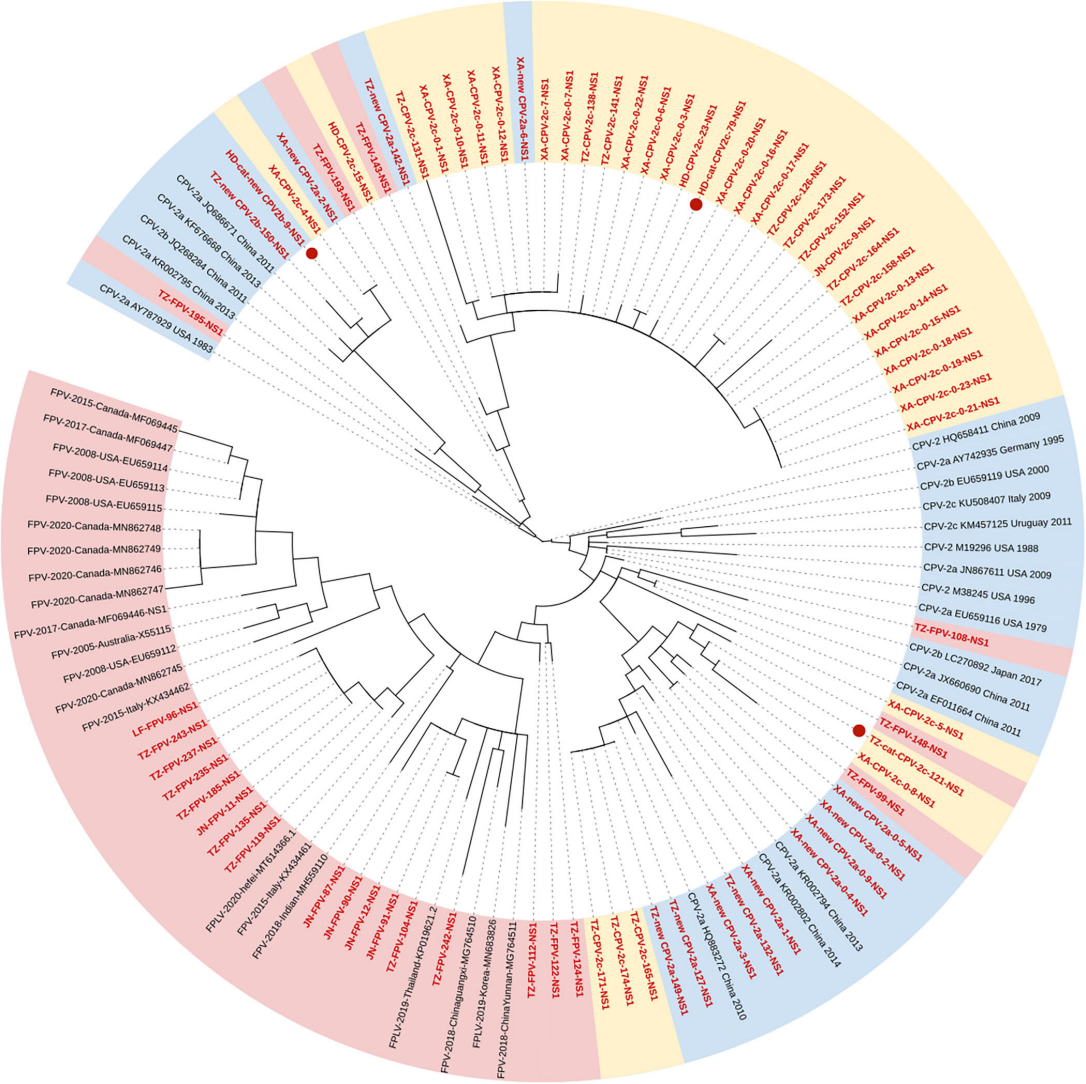
Phylogenetic tree based on a comparison of the non-structural (NS1) gene of canine parvovirus type 2 (CPV-2) and feline panleukopenia virus (FPV). Phylogenetic relationships were calculated using the maximum likelihood (ML) method. Gaps were handled by pairwise deletion and bootstrap values were calculated from 1,000 replicates. Strains analyzed in this study are marked in red and reference strains are marked in black. CPV-2 strains isolated from cats in this study are marked with “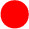.” The monophyletic clusters of new CPV-2a/2b are marked in blue (Clade IIA), the monophyletic clusters of CPV-2c are marked in yellow (Clade IIB), and the monophyletic clusters of FPV are marked in pink (Clade I).

Figure 2 shows the phylogenetic tree of the NS1 gene, and unlike the VP2 gene, the characteristic of clustering by genetic types was less supported (Figure 2). Seventeen FPV sequences formed the Clade I, new CPV-2a/2b, and CPV-2c strains clustered in Clades IIA and B, respectively. Meanwhile, two FPV strains and five CPV-2c strains were closed to Clade IIA, and another two FPV strains and two new CPV-2b strains were closed to Clade IIB. In addition, we found an individual clade comprising four strains with high homology (TZ-FPV-148, TZ-cat-CPV-2c-121, XA-CPV-2c-0-8, and TZ-FPV-99). The new CPV-2b and one CPV-2c strain infecting cats (HD-cat-newCPV2b-9 and HD-cat-CPV-2c-79) showed high homology with CPV-2 strains from dogs.

## Discussion

The VP2 protein is the main component of the virus capsid for determining antigenicity, host range, and hemagglutination (9). It has five important domains, Loop 1 (aa 50–100), Loop 2 (aa 200–250), Loop 3 (aa 300–350), Loop 4 (aa 400–450), and Loop 5 (aa 350–400) (23). Among them, different Loops perform specific functions. Loop 1, Loop 2, and Loop 4 not only constitute the spike of the triple folding (24) but also form the antigen A site with four key amino acid sites (93, 222, 224, and 426). Residue 440 is located on the top of the 3-fold spike as a main viral genetic site, which might result in the emergence of further genetic variants (25). Loop 3 not only forms the shoulder of the triple folding but also participates in the formation of the antigen B site, with the key amino acid sites 299, 300, and 302 (26).

In the results of CPV-2 VP2 gene alignment, CPV-2c strains harbored the key mutation sites with 370R and 440T, while new CPV-2a/2b genetic variants showed 370Q and 440A. Geng and colleagues reported that the VP2 protein of CPV-2c strains exhibited a unique Q370R substitution (27). Zhuang et al. also discovered that mutation Q370R was specific for all identified CPV-2c strains, and all the new CPV-2a strains showed the T440A mutation (28). This characteristic might help to distinguish CPV-2 genetic variants in more detail. In this study, CPV-2c was identified as the primary genetic variant in Beijing and Xiong'an of northern China. CPV-2c was first reported in Jilin province in 2010, and after that, it continued to spread from the north to the south of China (29) and it was reported as the dominant strain in many regions (30). Among FPV strains, 44.8% of strains had the A91S mutation. A91S is located in Loop 1 and T425A is located in Loop 4. These two mutation sites could influence the recognition of neutralizing antibodies against the antigen A site, maybe resulting in evading host immune surveillance. Based on the 3D structure prediction, A91S mutation extended the random coil of aa residues from 92–95 to 91–95 in FPV VP2 protein. Therefore, it is possible that the A91S mutation may also affect the receptor-binding ability (31). Further studies are then necessary to analyze *in vitro* any biological relevance of these mutations observed *in silico*.

Relatively enough data are available on the VP2 gene. But unfortunately, the current research on the NS1 gene is not enough to understand the corresponding protein structure and function, only three or five amino acid mutation sites were found. In previous studies, researchers evaluated the function of NS1 protein and described the potential location of functional domains, the origin of replication (ORI) binding (16–275aa), helicase (299–486aa), and transactivation (600–667aa) functional domains (32). In this study, we highlighted the role of amino acid positions 60 and 630, which are located on the ORI binding and transactivation functional domains, respectively. Transactivation can increase the rate of gene expression (33). Residue 443 putatively lies in the β3-sheet of the Walker motif B of the helicase domain, and residues 350 and 544–545 are located between the α5- and α6-helices close to the α11-helix of the same domain (32). Helicases open the complementary double strands of DNA using energy derived from ATP hydrolysis to obtain single strands; therefore, these functional domains play an important role in early viral replication and DNA assembly (34). Nevertheless, most mutation sites tend to gather in these functional domains, which might influence the host range and virulence. Researchers considered that amino acid site 248 can be used to distinguish CPV-2 and FPV (11). In the case of a nucleotide change in the second base of the codon (c743t), FPV showed 248T (act), while CPV-2 showed 248I (att). By contrast, our research found that all CPV-2 strains showed 248I (att), while among FPV strains, 78.3% of strains showed 248T (act) and five strains (TZ-FPV-99, TZ-FPV-108, TZ-FPV-143, TZ-FPV-148, and TZ-FPV-193) showed 248I (att). This indicated that neither NS1-248 aa residue distinguished FPV from CPV-2.

At present, although various hypotheses explaining its derivation and sudden emergence have been proposed, opinions on the origin of CPV-2 were still not conclusive. The most widely accepted hypothesis for its emergence is that CPV-2 was derived from FPV in cats or FPV-like viruses in wild animals by a natural genetic mutation (35). Truyen et al. reported that ~5% of isolates from domestic cats in Germany and the USA were either CPV-2a or CPV-2b, while the original CPV-2 could not replicate in feline tissues (36). The pathogenicity of the CPV-2 genetic variants was milder and could replicate only poorly in cats, with mild clinical signs, such as leukopenia and poor growth. Subsequently, it was reported that the genetic variants of CPV-2,−2a,−2b, and−2c have acquired the feline host range, and were able to infect and replicated efficiently in cats and some wildlife (37–39). Our results revealed that CPV-2 genetic variants were circulating in dogs and, with low prevalence in cats, which demonstrated the need for continued surveillance of FPV and CPV-2 infections considering the key amino acid sites in both structural and non-structural proteins.

## Conclusion

The epidemiology of CPV-2 and FPV was assessed in northern China from 2019 to 2020, and CPV-2c strains were the dominant CPV-2 genetic variant among dogs. Within the summarized mutation sites of NS1, amino acid positions 60, 630, 443, 544, and 545 maybe play an important role in genomic replication and virion packaging. Further studies are then necessary to elucidate the relevance of these mutations in the biological properties of the NS1 protein functional domains. Besides, one new CPV-2b strain and two CPV-2c strains were collected from domestic cats.

## Data availability statement

The datasets presented in this study can be found in online repositories. The names of the repository/repositories and accession number(s) can be found in the article/Supplementary material.

## Ethics statement

The animal study was reviewed and approved by the Institute of Animal Sciences, Chinese Academy of Agricultural Sciences.

## Author contributions

XC: resources. SL: investigation and writing—original draft. YH: formal analysis. TQ: supervision and project administration. GZ, YL, JW, and WL: writing—review and editing. All authors contributed to the article and approved the submitted version.

## Funding

This work was supported by the Agricultural Science and Technology Innovation Program (Grant No: ASTIP-IAS15).

## Conflict of interest

The authors declare that the research was conducted in the absence of any commercial or financial relationships that could be construed as a potential conflict of interest.

## Publisher's note

All claims expressed in this article are solely those of the authors and do not necessarily represent those of their affiliated organizations, or those of the publisher, the editors and the reviewers. Any product that may be evaluated in this article, or claim that may be made by its manufacturer, is not guaranteed or endorsed by the publisher.
