# Supplementary material for: Characterization of the VP2 and NS1 genes from canine parvovirus type 2 (CPV-2) and feline panleukopenia virus (FPV) in Northern China
Source: Front Vet Sci. 2022 Nov 28;9:934849. doi: 10.3389/fvets.2022.934849 (PMC9742280; doi:10.3389/fvets.2022.934849)
Supplement: Supplementary file 1 [file Table_1.DOCX]

| **VP2** | | | | **NS1** | | | |
| --- | --- | --- | --- | --- | --- | --- | --- |
| **FPV** |  | **CPV-2** |  | **FPV** |  | **CPV-2** |  |
| EU498681  MH165482  MT274377.1  MN683826  KP019621.2  HQ184204.1  MK671181.1  HQ184196  EF988660  EU221281.1  MT274378.1  MK671177  MK671187,1  MK671188,1  FJ440712.1  MK671185.1  MK301396.1  MT614366.1 | | LC270892  AY742955  FJ197835  DQ340433  AB054213  AY869742  AB120727  FJ222821  KM236569  FJ005235  KU508407  KM457125  KJ674820  KF676668  KR002802  KR611488  MK332005  KJ674818  JX660690  JQ686671  KU244254  KT156832  MK332007  MK332001  KY937650  KT162005  KR611522  MF467229 | | MF069445  MF069447  EU659114  EU659113  EU659115  MN862748  MN862749  MN862746  MN862747  MF069446  X55115  EU659112  MN862745  KX434462  MT614366.1  KX434461  MH559110  KP019621.2  MG764510  MN683826  MG764511 | | HQ883272  KR002802  KR002794  EF011664  JX660690  LC270892  EU659116  M38245  JN867611  M19296  KM457125  KU508407  EU659119  AY742935  HQ658411  JQ686671  KF676668  JQ268284  KR002795  AY787929 | |

Table S1. The reference sequences of the NS1 gene and the VP2 gene were obtained from the GenBank database for constructing the phylogenetic trees.
